# Preoperative Doppler Ultrasonography Allen Test for Radial Forearm Free Flap in Oral Cancer Reconstruction: Implications in Clinical Practice

**DOI:** 10.3390/jcm10153328

**Published:** 2021-07-28

**Authors:** Manuel Tousidonis, José Ignacio Salmerón Escobar, Santiago Ochandiano Caicoya, Carlos Navarro Vila, Ignacio Navarro Cuéllar, Alberto Díez Montiel, Ana María López López, Carlos Navarro Cuéllar

**Affiliations:** Maxillofacial Surgery Department, Hospital General Universitario Gregorio Marañón, C/Doctor Esquerdo 26, 28007 Madrid, Spain; jisalmeron@telefonica.net (J.I.S.E.); sochandiano@hotmail.com (S.O.C.); cnavarro.vila@clinicanavarrovila.com (C.N.V.); nnavcu@hotmail.com (I.N.C.); diezmontiel@gmail.com (A.D.M.); anitalopez@gmail.com (A.M.L.L.); cnavarrocuellar@gmail.com (C.N.C.)

**Keywords:** radial forearm flap, Allen test, oral reconstruction, Doppler ultrasonography, donor site morbidity

## Abstract

The Radial Forearm Free Flap (RFFF) is one of the most widely used microsurgical flaps for intraoral reconstruction. Although the Clinical Allen Test (CAT) is the most widely used preoperative diagnostic method with which to study the distal patency of the hand prior to the use of RFFF, several authors have reported potentially preventable serious vascular complications. This study included 87 consecutive patients with cancer of the oral cavity and RFFF as the flap of choice who were treated between 2010 and 2020, and compares the results of the Clinical Allen Test (CAT), the Doppler Allen Test (DAT) and the Surgical Allen Test (SAT). The preoperative vascular study found vascular abnormalities severe enough for the surgical team to change the preoperative flap of choice in 39% of patients. The Kappa index showed a weak concordance between the CAT and DAT. The study reflected a total concordance in the preoperative results of the Doppler study and the intraoperative results of the SAT. Due to its excellent agreement with SAT, the DAT would be the preoperative test of choice in patients who are candidates for RFFF. This study of vascular mapping tests with Doppler is intended to inform therapeutic decisions and present methods to gain information that cannot be obtained by physical examination alone.

## 1. Introduction

Cancer of the oral cavity is one of the most common malignancies; the main etiological factors are tobacco and alcohol use. The standard of care is primary surgical resection with or without postoperative adjuvant therapy. Restoration of form and function after ablative cancer surgery is the ultimate goal of treatment, and is achieved by choosing the appropriate reconstructive procedure. Since the development of microvascular free tissue transfer techniques, surgeons have rapidly expanded their ability to resect extensive disease and provide multiple reconstructive options. Among free flaps, the radial forearm free flap (RFFF), first described by Yang GF et al. [1], has become one of the most used in oral cancer reconstruction [2] due to its versatility, ease of harvesting using a two-team approach, as well as providing a thin, flexible, sensitive skin paddle which is of sufficient size to reconstruct medium-sized defects with a long vascular pedicle. The availability of other tissues such as bone, nerve, and tendon has increased the applications of the flap. The success rate has been reported to be approximately 97% [3]. The RFFF vasculature is thought to be resistant to atherosclerotic disease. However, RFFF is associated with significant donor site morbidity; ischemic hand complications (IHCs) are the most feared postoperative morbidities [4,5,6,7,8,9]. 

Harvesting RFFF involves the removal of the radial artery. The most important requirement for harvesting RFFF is an intact palmar arch that provides perfusion of the hand by the ulnar artery [10,11]. The use of the nondominant arm as a donor site is preferred. If the ulnar and radial anastomoses are missing, a reduction in circulation to the hand would be expected, especially if the removed artery is the dominant one. Several studies have demonstrated the existence of vascular abnormalities that cannot be detected preoperatively by physical examination, the presence of which can jeopardize the viability of RFFF. 

In common clinical practice, the permeability of the palmar arch is evaluated by the Clinical Allen Test (CAT) [10,11]. Once pressure on the ulnar artery is relieved (while holding continued pressure on the radial artery), one should see a return of waveform and strong oximetry signal within 5 s (negative CAT). A negative CAT is accepted as a condition for raising the radial forearm flap. A positive CAT precludes surgery. A substantial delay in return of the waveform or a dampened waveform, however, should alert the surgeon to the possibility of an incomplete arch. Allen originally described his test for diagnosis of thromboangeitis obliterans of the ulnar artery [12]. The reported sensitivity and specificity of the Clinical Allen test are 54.5 and 91.7%, respectively [13], but these values do not provide a clinical image or information about vascular dynamics. It is established in common practice that CAT can ensure that the hand will remain well-perfused after division of the radial artery [14], but critical ischaemia of the hand has been described in patients with negative CAT [6,7,8,9] and, despite the publications, many microsurgery teams worldwide consider that preoperative vascular imaging is rarely needed [13,14,15,16]. The most sensitive and specific test is the direct intraoperative visualization and clamping of the radial artery (Surgical Allen Test, SAT) before the radial artery ligation in RFFF harvesting. The SAT is not a preoperative test. The SAT with pulse oximeter control should be performed in all cases before ligation of the radial artery.

Computer tomography angiography (CTA), magnetic resonance angiography (MRA) and Doppler ultrasonography (DUS) are the most relevant vascular mapping tests in reconstructive microsurgery; their use has been shown to increase the success of free flap reconstruction, decrease surgery time and reduce the morbidity of the donor area [14]. With respect to Doppler, CT and MR are more expensive and less versatile because they cannot be performed at the bedside and, in the case of CT, the patient must be irradiated. No vascular mapping test is considered the gold standard in the preoperative study of the RFFF. The main limitation for the use of CTA and MRA as preoperative imaging tests in RFFF is that they do not allow the Allen test to be performed. DUS, in contrast, provides information of vascular anatomy, ultrasound waveforms and flow velocities, and allows the Allen test to be performed (Doppler Allen Test, DAT) [17,18]. DAS is more sensitive and specific than CAT. With DUS, it is possible to avoid CAT false negative results from incomplete compression of the radial artery and hyperextension of the wrist [19]. 

The objective of the study was to analyze the findings of preoperative Doppler ultrasonography with the Allen Test in a series of patients whose reconstruction of choice was the RFFF, and to compare the results obtained in the DAT with those of the CAT and SAT, evaluating the concordance between the three measures and discussing their clinical implications. We hypothesize that DUS should be considered the preoperative vascular imaging test of choice before RFFF harvesting; however, no study in the literature to date has focused on the clinical implications of DUS prior to RFF specifically. 

## 2. Materials and Methods

This study was designed to collect data retrospectively regarding preoperative findings on vascular anatomy with Doppler ultrasonography and its relationship with clinical outcomes in patients with oral cancer whose preoperative flap of choice for oral reconstruction was the radial forearm free flap (RFFF). The inclusion criteria of the patients were: diagnosis of oral cancer, surgery as the treatment of choice, radial free flap as the reconstruction of choice for the oncological defect, preoperative study with CAT and DAS in all patients, Karnofsky index equal to or greater than 40, and absence of metastatic disease. The study included 87 consecutive patients with cancer of the oral cavity and RFFF as the flap of choice who were treated between 2010 and 2020 in the Oral and Maxillofacial Department of the Hospital General Universitario Gregorio Marañón, Madrid, Spain. The study was conducted according to the guidelines of the Declaration of Helsinki and endorsed by the Hospital Ethics Committee at Gregorio Marañón General Hospital, Madrid, Spain (protocol code maxilohgugm 09/2020). Postoperative clinical notes were reviewed for complications. Informed consent was obtained from all patients.

Preoperative evaluation included CAT and DAS in all patients. A Clinical Allen Test and Doppler Allen Test were performed in the standard procedure and categorized according to flow as sufficient (<5 s), indeterminate (6–10 s), or insufficient (>10 s). DAS was performed using DUS by the same senior vascular surgeon in all cases.

A Surgical Allen Test (SAT) was performed intraoperatively by clamping the radial artery and ensuring that all perfusion of the hand was via the ulnar artery using a pulse oximeter. This assessment was considered the criterion standard of sufficient collateral flow before ligation of the radial artery.

All patients underwent tumor resection, neck dissection (unilateral or bilateral), and microvascular reconstruction with RFFF of the nondominant hand or other flap when preoperative DAS contraindicated its use. The sex and age of the patients, anatomical locations of the tumors and subsequent defects, clinical stage of disease, size of the flap, duration of free flap harvesting, unilateral or bilateral neck dissection, tracheostomy, smoking history, histology, primary or secondary reconstruction, duration of surgery, cardiovascular risk factors, and donor site treatment and complications are listed in Table 1. Dominant and nondominant hand CAT, and dominant and nondominant hand DAT results are listed in Table 2. Changes in the decision of the flap side and contraindication or not to the use of RFFF are listed in Table 3. The dual blood supply to the hand and CAT and DAT was recorded in all patients. The SAT was recorded in all cases of RFFF harvesting. In all cases, flap pedicles were long enough to reach the recipient’s neck vessels. The donor site defect was closed using a split-thickness skin graft harvested from the groin region and secured with nonabsorbable sutures. The recipient arteries and veins (1 or 2, when the cephalic vein together with a vena comitans were used) were collected in this study. Postoperative data were reviewed to assess complications.

### Statistical Analysis

The statistical analysis was conducted using SPSS version 25.0 (SPSS Inc., Chicago, IL, USA). The data were recorded as the mean and standard deviation (SD) values. The normality of the distribution was previously tested by Saphiro-Wilk test. A concordance study was performed using the kappa index between the results of the CAT, DAT and SAT to evaluate the strength of the agreement between the preoperative study methods (CAT and DAT) in order to compare them with the gold standard (SAT). *p*-values less than 0.05 were considered statistically significant. 

## 3. Results

Eighty-seven patients with a diagnosis of oral cancer met the inclusion criteria and underwent surgery during the study period. The mean follow-up time was 38.5 months (range: 1–110 months). The male:female ratio was 2:1. The time taken to assess the limb use duplex was 19 min (range: 13–27). The mean (range) diameter of the radial artery at the elbow was 3.13 mm (range: 1.2–5.1) and at the wrist 2.19 mm (range 0.9–4.1). Vascular abnormalities were recorded, and 34 patients (39%) were deemed serious enough for the surgical team to change the preoperative flap of choice (RFFF of the nondominant hand) by using the radial of the dominant hand (24 cases) or perform an alternative flap (10 cases). Loss of the RFFF occurred in one case (1.14%), reconstructed in a second surgical procedure with a FAMM flap. A different flap had to be performed in 10 patients, either due to loss of the previous radial flap (one case) or because the preoperative mapping tests contraindicated the use of the radial flap bilaterally (nine cases). An anterolateral thigh (ALT) flap was used in three patients, a FAMM flap in three patients, a pectoralis myocutaneous pedicle flap in two patients, a temporalis muscle flap in one, and a nasolabial flap in one case. A total of 80 cases (92%) were reconstructed with microsurgical flaps (77 RFFF, 3 ALT), and 7 (8%) with pedicled flaps. The recipient arteries of the microsurgical anastomoses were: facial artery (46 cases, 57.5%), superior thyroid artery (22 cases, 27.5%), lingual artery (7 cases, 8.75%), external carotid artery (3 cases, 3.75%), and transverse cervical artery (2 cases, 2.5%). In the case of venous anastomoses, two venous anastomoses were performed in cases in which the vascular anatomy was favorable. The recipient veins of the microsurgical anastomoses were: thyrolinguofacial trunk (65 cases, %), external jugular vein (53 cases, %), internal jugular vein (7 cases, 8.75%), facial vein (6 cases, 7.5%), superior thyroid vein (6 cases, 7.5%), and lingual vein (1 case, 1.25%). A total of 68 patients received adjunctive treatment with radiation therapy. No patient developed ischemia of the hand.

Documentation of the CAT and DAT were available in all patients. The disagreement between the clinical findings of the Allen Test (CAT) and the findings of the Allen Test with Doppler (DAT) without taking into account the indeterminate cases occurred in 16 upper limbs (9.2%) out of a total of 174. If we consider the doubtful or indeterminate cases, errors between CAT and DAT occurred in 47 upper limbs (27%) out of a total of 174 (Table 2). It is important to consider that in 13 patients in which the initial CAT exploration was negative (which would allow the use of RFFF), the Doppler ultrasonography exploration had a positive DAT, which contraindicated the use of RFFF and avoided serious ischemic complications. In the 14 cases of CAT +, the Doppler usually confirmed the impossibility of using the RFFF of the upper limb studied (12/14 cases). In cases of indeterminate CAT, the Allen Test with Doppler is essential to facilitate decision making between the various reconstructive possibilities (nondominant hand RFFF, dominant hand RFFF, or other reconstructive options). In all patients, SAT was performed with pulse oximetry control before ligating the radial artery. The agreement between the DAT and SAT was 100%. No patient with a negative Doppler study (DAT-) suffered a decrease in distal hand oxygen saturation after SAT. Therefore, no intraoperative changes had to be made in the flap of choice. 

When performing the concordance study between the CAT and DAT, the Kappa coefficient or Kappa index showed a weak concordance strength. The Kappa index when comparing the CAT and the DAT was 0.340, with a standard error of 0.107 for the study sample (95% confidence interval: 0.130–0.551). When comparing the DAT and SAT, the Kappa index was 1.00, with total agreement in the preoperative results of the Doppler study and the intraoperative results of the SAT.

## 4. Discussion

The RFFF is one of the most commonly used flaps in oral reconstruction, providing thin, pliable soft tissue and a high transfer success rate. RFFF success was 98.86% in our series, similar to the results reported by other reconstructive surgery groups. The RFFF is the flap of choice for multiple oncological defects of the oral cavity, but other reconstructive options can be used (both pedicled flaps and microsurgical flaps). As there are other therapeutic alternatives, we must minimize the risk of associated morbidity. Ischemic complications of the hand in RFFF harvesting are the most feared complications, and have been extensively described in the scientific literature [4,5,6,7,8,9]. In our series, we did not encounter any major complications in the donor area, and no ischemic event occurred during the study follow-up period. Several authors [20] declared that the use of the radial artery for microsurgical reconstruction could cause a deterioration of the distal vascularization of the hand. Complete acute ischemia of the hand has also been described in a patient with normal CAT [6]. The main limitations of physical examination are the impossibility of diagnosing vascular anomalies, and that negative CAT does not ensure distal perfusion of the hand. It is not clear when reviewing the scientific literature that a positive or pathological DAT will produce real consequences for the patient in terms of changes in the distal perfusion of the hand, but it does provide relevant clinical information on possible risk situations due to reversal of vascular flow. Studies in experimental animals would be necessary to study acute and chronic changes in the distal vascularization of the hand in situations of pathological DAT. As a preoperative test in the RFFF, the DAT provides vascular information on the distal perfusion of the limb, relevant in situations of doubtful CAT, and especially considering the possibility of being able to use the contralateral side or perform another reconstruction flap.

The use of high-resolution vascular mapping preoperative tests on microsurgical flaps has been shown to decrease intraoperative complications in flap dissection [21], decrease overall surgical time and flap harvesting time [22], facilitate dissection and avoid serious vascular complications [23] in patients with vascular abnormalities that could not be diagnosed without such vascular tests or only with physical examination [22,23,24]. There is published evidence on the improvement of results when performing preoperative vascular mapping studies with MRA and CTA in several microsurgical flaps: fibula flap [24,25,26,27], deep inferior epigastric perforator flap [21,22,23,28,29,30,31] and other perforator flaps. The evidence for the use of preoperative vascular mapping tests in RFFF is scarce, with contradictory results. The most widespread clinical practice prior to harvesting a RFFF is to perform CAT without any preoperative vascular mapping test [14]. Another problem to be solved in the use of the preoperative vascular test in RFFF is the type of test. The ideal preoperative vascular test for RFFF would be one that allows the diagnosis of vascular abnormalities of the upper limb and can also confirm the adequacy of the ulnar artery to distally perfuse the hand. MRA and CTA are the most widely used tests for vascular preoperative study in reconstructive microsurgery. Both are high-resolution tests which are suitable for the three-dimensional diagnoses of vascular alterations; however, they do not allow a dynamic evaluation of the distal irrigation of the hand with the Allen Test. The only test that provides simultaneously three-dimensional vascular anatomy data and the possibility of performing the Allen Test is Doppler Ultrasonography, which is the most sensitive and specific diagnostic test to assess the distal patency of the hand, simulating the ligation of the radial artery performed in the RFFF. Therefore, it should be considered as the preoperative test of choice in the RFFF, being the only preoperative test that can simulate radial artery ligation, with a 100% correlation with the gold standard (SAT, Surgical Allen Test) in our series. The DSA is a less relevant test at present due to its associated morbidity and the fact that it does not provide additional information to CTA or MRA.

Vascular dominance of the hand is a subject under discussion. According to De Vicente et al. [15], the radial artery is the dominant artery in the distal vascularization of the forearm. Dost and Rudofsky [32] observed dominance of the radial artery in 6 of 11 patients, whereas in the other 5, the ulnar artery was dominant. Hirai [33] reported that 26% of patients had radial dominance, 24% ulnar dominance and 50% had no dominant vessel. Fuhrman [34] published similar data. Haerle et al. [35] stated that the ulnar artery was dominant in the elbow area while the radial artery was dominant in the wrist. In their anatomical study of the superficial palmar arch, Bilge et al. [36] concluded that a complete palmar arch is found in approximately 85% of hands, an essential requirement for performing RFFF. Vascular anomalies of the upper limb are relatively frequent, and are asymptomatic in most cases. Physical examination is insufficient for the diagnosis of most vascular alterations of the distal vascular system of the upper limb. A large number of vascular alterations have been described. Coleman and Anson [37] demonstrated that there were no anastomosis between the superficial palmar arch and the radial artery in 12–60% of cases, and between the deep palmar arch and the ulnar artery in 54%. McCormack et al. [38] found anomalies of the brachial and radial arteries in 15% of cases according to their anatomical studies, similar to the percentage of vascular anomalies in our series, which is 12.7% of the total upper limbs studied. Uglietta and Kadir found abnormalities in 9% of radial arteries in arteriographic examination [39]. Porter and Mellow [40] described an entirely absent radial artery. Bell et al. [41] described in 2011 the superficial ulnar artery as a contraindication to RFFF. Acaturk et al. [42] described an accessory branch or the radial artery coursing superficially, naming it the superficial radial artery. Radial artery duplication is another well documented anomaly; Li et al. [43] documented duplication in 0.1% of 1400 coronary angiography patients sampled via ultrasound. The radial artery in carpal tunnel is a clinical variation with important implications during RFFF harvesting [44,45,46,47,48]. An aberrant vessel originating at the axillary artery and parallel to the brachial artery on its distal course was described by Mordick [47]. Funk et al. [48] described a radial artery passing deep the pronator teres rather than superficial. The absence of the ulnar artery has been estimated to occur in at least 0.015% of cases [49]. A case of an anomalous radial artery arising from the thoracoacromial trunk was described by Loukas et al. [50]. The most described vascular abnormality of the radial artery is a high radial origin [51,52,53]; this particular vascular anomaly does not impact RFFF. Madaree and McGibbon discovered a radial artery without any fasciocutaneous perforators to the forearm [54]. In our series, the most frequent vascular anomaly is high radial origin (3.2%). Whenever possible, the RFFF of choice is that of the nondominant hand. Preoperative dynamic vascular findings with Doppler Ultrasonography preclude the flap of choice in 34 of the 87 patients (39%), forcing the use of the RFFF of the dominant hand in 24 surgeries and contraindicating the use of the RFFF in 9 patients. The clinical implications of these findings are relevant because they affect the reconstructive therapeutic algorithm and reduce potentially avoidable serious vascular complications.

Although the DAT has a 100% match with the SAT, it remains unclear to what extent a pathological DAT also results in real consequences for the patient in terms of changes in distal hand perfusion, and future studies are needed to compare the findings of DAT and fluorescent angiography both preoperatively and postoperatively to monitor evolutionary changes in distal hand perfusion in patients who have undergone RFFFF. 

The main weaknesses of the study are the absence of a control group, that patients were not randomly assigned and it was a monocentric study with patients with the same pathology (oral cancer), which reduces external validity. 

## 5. Conclusions

Ischemic hand complications after RFFF reconstruction in patients with oral cancer are an avoidable complication which is very rare in patients with normal CAT. The agreement between the CAT and DAT was low in our series; however, the DAT had a 100% agreement with the SAT. Due to its excellent agreement with SAT, the Doppler would be the vascular preoperative test of choice for patients eligible for reconstruction with a RFFF, mainly in cases with doubtful or positive CAT, as it may help in therapeutic decision-making and provide information that cannot be obtained by physical examination and CAT.

## Figures and Tables

**Table 1:** Baseline characteristics of 88 patients.

Characteristics	*n*	%
Age (years), mean ± SD (range)	58.3 (12.1)	
Sex		
Female	59	67.8
Male	28	32.2
Site		
Tongue	42	48.3
Floor of the mouth	12	13.8
Gingiva	11	12.6
Buccal	10	11.5
Oropharynx	7	8.0
Palate	5	5.7
Histology		
Squamous cell carcinoma	78	89.6
Adenoid cystic carcinoma	4	4.6
Adenocarcinoma	2	2.3
Chondrosarcoma	1	1.1
Malignant Schwannoma	1	1.1
Carcinoma ex pleomorphic adenoma	1	1.1
Reconstruction		
Primary	85	97.7
Secondary	2	2.3
Neck dissection		
No	7	8.1
Unilateral	29	33.3
Bilateral	51	58.6
Tracheostomy		
Yes	81	93.1
No	6	6.9
Stage		
I	4	4.6
II	22	25.3
III	14	16.1
IV	47	54.0
Smoking habit		
Yes	73	83.9
No	14	16.1
Donor site closure		
No	76	87.3
Loss of graft (partial)	7	8.1
Tendon exposure	4	4.6
Size of flap (cm2), mean ± SD (range)	37.34 ± 7.24 (15–99)	
Time taken to raise the flap (min), mean ± SD (range)	73.76 ± 25.22 (46–127)	
Duration of surgery (min), mean ± SD (range)	645.37 ± 118.16 (303–974)	
Cardiovascular risk factors		
0	23	26.4
1	14	16.1
2	23	26.4
>2	27	31.1

**Table 2:** Correlation between Clinical Allen Test (CAT) and Doppler Allen Test (DAT) in dominant and nondominant hand.

Dominant Hand				
	**DAT +**	**Indeterminate**	**DAT −**	**Total**
CAT +	4	1	1	6
Indeterminate	7	0	10	17
CAT −	4	0	60	64
Total	15	1	71	87
**Nondominant Hand**				
	**DAT +**	**Indeterminate**	**DAT –**	**Total**
CAT +	7	0	1	8
Indeterminate	9	0	5	14
CAT –	9	0	56	65
Total	25	0	62	87

**Table 3:** Changes in the decision of the flap of choice due to vascular preoperative studies.

Preoperative Vascular Findings	*n*	Use of the Flap of Choice	Type
Normal	53	Yes	Nondominant RFFF
Pathological findings	34	No	Other (24 dominant RFFF; 9 contraindication RFFF)

## Data Availability

The data presented in this study are available on request from the corresponding author. The data are not publicly available due to data protection regulations.
